# DRD2, DRD3, and HTR2A Single-Nucleotide Polymorphisms Involvement in High Treatment Resistance to Atypical Antipsychotic Drugs

**DOI:** 10.3390/biomedicines11072088

**Published:** 2023-07-24

**Authors:** Antonio Del Casale, Maurizio Simmaco, Martina Nicole Modesti, Clarissa Zocchi, Jan Francesco Arena, Irene Bilotta, Alessandro Alcibiade, Giuseppe Sarli, Lorenzo Cutillo, Giulia Antonelli, Enrico La Spina, Ottavia De Luca, Robert Preissner, Marina Borro, Giovanna Gentile, Paolo Girardi, Maurizio Pompili

**Affiliations:** 1 Department of Dynamic and Clinical Psychology and Health Studies, Faculty of Medicine and Psychology, Sapienza University of Rome, 00185 Rome, Italy; antonio.delcasale@uniroma1.it; 2 Unit of Psychiatry, ‘Sant’Andrea’ University Hospital, 00189 Rome, Italy; 3 Department of Neuroscience, Mental Health and Sensory Organs (NESMOS), Faculty of Medicine and Psychology, Sapienza University of Rome, 00185 Rome, Italy; 4 Unit of Laboratory and Advanced Molecular Diagnostics, ‘Sant’Andrea’ University Hospital, 00189 Rome, Italy; 5 Structural Bioinformatics Group, Institute for Physiology, Charité—University Medicine Berlin, 10115 Berlin, Germany

**Keywords:** antipsychotic agents, dopamine receptors, 5-hydroxytryptamine receptors, drug resistance, pharmacogenomic variants, precision medicine

## Abstract

Background: The objective of this study was to investigate the *DRD2* rs1800497, rs1799732, rs1801028, *DRD3* rs6280, and *HTR2A* rs6314, rs7997012, and rs6311 single-nucleotide polymorphism (SNP) correlations with resistance to second-generation antipsychotics (SGAs) in a real-world sample of patients with treatment-resistant mental disorders. Methods: We divided 129 participants into a high treatment resistance (HTR) group (current treatment with two SGAs, or clozapine, or classic neuroleptics for a failure of previous SGAs trials) and a low treatment resistance (LTR) group (current treatment with one atypical antipsychotic). We used Next-Generation Sequencing on DNA isolated from peripheral blood samples to analyze the polymorphisms. We performed logistic regression to search for predictors of HTR membership. Results: A diagnosis of schizophrenia significantly predicted the HTR membership compared to other diagnoses. Other predictors were the *DRD3* rs6280 C|T (OR = 22.195) and T|T (OR = 18.47) vs. C|C, *HTR2A* rs7997012 A|G vs. A|A (OR = 6.859) and vs. G|G (OR = 2.879), and *DRD2* rs1799732 I|I vs. D|I (OR = 12.079) genotypes. Conclusions: A diagnosis of schizophrenia and the *DRD2* rs1799732, *DRD3* rs6280, and *HTR2A* rs7997012 genotypes can predict high treatment resistance to SGAs.

## 1. Introduction

Antipsychotics have entirely changed our perspective on psychosis treatment since they were discovered [1,2]. These drugs are currently included in guidelines for disorders such as schizophrenia spectrum disorders (SSD) [3,4], bipolar disorder (BD) [5], and mood disorders [6,7]. Also, there is evidence for their effectiveness in the treatment of other illnesses, including obsessive–compulsive disorder (OCD) [8], post-traumatic stress disorder with psychotic features (PTSD-SP) [9], neurocognitive disorders [10], autism spectrum disorders (ASD) [11], insomnia [12], and other mental disorders with psychotic symptoms.

However, even though some of the primary antipsychotics’ achievements concern SSD, and more than 60 antipsychotics are available presently, up to 20–30% of people affected are resistant to treatment, defining a clinical entity called treatment-resistant schizophrenia (TRS) [13]. In 2017, the Treatment Response and Resistance in Psychosis (TRRIP) group found no consistency between the TRS definitions. Hence, TRS was defined by at least two failed antipsychotic trials of sufficient dosage and duration [14]. Among available antipsychotics, clozapine is indicated in the first place when TRS occurs [4] and, as the most effective, represents the gold-standard treatment [15]. Lately, many studies have focused on clozapine’s evidence base: on the one hand, its superiority was confirmed. On the other hand, further studies are needed to address this drug more precisely, given its tolerability [16]. In this direction, pharmacogenomics has been considered a possible option to identify a priori the clinical populations more likely to respond adequately to a trial [17]. Nevertheless, 30–60% of patients will show an insufficient response, suggesting that drug responsiveness/resistance is unlikely to represent the key to understanding this condition [18]. Our knowledge of this disorder is still limited, and exploring the underpinning mechanisms will be a fundamental challenge to developing new adequate treatment protocols.

Drug resistance is a central topic in research about major depressive disorder (MDD). Less than 50% of responding patients will experience remission since at least two or more symptoms persist [19]. In analogy with TRS, over ten definitions of treatment-resistant depression (TRD) can be found across literature [20], and a lack of consistency is still present from both methodological and semantic points of view. Lately, concern about the validity of this standard definition, which may not fit with real-world practice [21], has highlighted a possible epistemological confusion that hinders research in drug-resistant disorders. The regulatory description of TRD provided by the Food and Drug Administration (FDA) [22] requires the failure of more than two treatments, adequate in dosage and duration, with at least two different ADs. Currently, the most common strategies to treat TRD are increasing the dosage, augmentation, or switching to a new antidepressant [23]. According to CANMAT [24], APA [25], and NICE [6] guidelines, the use of second-generation antipsychotics (SGAs) is a possible augmentation strategy, especially in specific subtypes of depression (with psychotic features, mixed features, and sleep disturbances) [24]. In this sense, among other compounds, there is more and more evidence for using SGAs [26,27], which are a valid option even when compared to lithium and esketamine [28].

It is a common clinical experience for a drug to show variability in the response, not only within the same diagnostic category but also in a person-dependent way. Among many factors possibly underlying this variance, drug–drug and drug–gene interactions are the most important [29]. Pharmacogenomics (PGx) represents one of the leading research areas in precision medicine and focuses on drug response by investigating patients’ genetic makeup. At present, more than 10% of medications approved by the FDA provide in their labels PGx information concerning potential adverse drug reactions (ADRs) and guidelines for adjusting the drug dose or switching [30]. However, full/stable remission rates among patients with major psychiatric disorders are still unsatisfying. Relevant genetic variants could be used as pharmacogenomic biomarkers for drug treatment [29] to guide prescription and overcome the “trial-and-error” method. In this sense, a significant effort has been focused on genetic polymorphisms encoding drug targets such as receptors (*HTR2A*, *HTR2C*, *DRD2*), the serotonin transporter (*SCL6A4*), transporter genes (mainly ABCB1), and drug-metabolizing enzymes (CYP450) [29].

For example, different *DRD2* (rs1800497, rs1799732), *DRD3* (rs6280), and *HTR2A* (rs7997012) single-nucleotide polymorphisms (SNPs) were involved in olanzapine effectiveness [31] and in the occurrence of extra-pyramidal symptoms (EPS) [32]. Furthermore, on the one hand, rs1799732 has been associated, in particular, with risperidone response [33]. On the other hand, rs1800497, which robustly influences the striatal dopamine receptor availability [34], was linked to the occurrence of akathisia induced by SGAs [35], the severity of EPS in CYP2D6 extensive metabolizers [36], dopamine hypersensitivity psychosis, and treatment resistance in patients affected by schizophrenia [37]. In this regard, another *DRD2* SNP (rs1801028) showed an association with risperidone efficacy on negative symptoms in a Han Chinese sample of patients [38]. Among *HTR2A* SNPs, rs6314 was involved in clozapine response [39,40], and rs6311 in schizophrenia [41]. Furthermore, the rs6314 was involved in functional changes in the prefrontal cortex and olanzapine response, showing that the T allele was associated with abnormalities in receptor expression and lower improvement of negative symptoms during treatment [42]. Among young patients affected by autism, the carriers of the *Gly* allele for a *DRD3* SNP (rs6280) showed a significantly better response to risperidone, widely employed in this field [43].

To the best of our knowledge, there is still a lack of research regarding specifically general resistance to antipsychotic agents since existing studies frequently focus on schizophrenia and mostly regard the involvement of polymorphisms in genetic risk, drug response, effectiveness, and tolerance. However, considering the available evidence, we hypothesized that specific genotypes could be related to high treatment resistance towards SGAs and predict a higher or lower risk of treatment resistance.

This study focused on the *DRD2* rs1800497, rs1799732, rs1801028, *DRD3* rs6280, and *HTR2A* rs6314, rs7997012, rs6311 polymorphisms to investigate possible correlations between resistance to antipsychotic agents and genotypes in a real-world sample of patients affected by various difficult-to-treat mental disorders.

## 2. Methods

An observational, retrospective study was conducted during 2018–2022 at the Centre of Personalized Medicine and Service of Personalized Mental Health and Pharmacogenomics, Unit of Psychiatry, Sant’Andrea University Hospital, Sapienza University, Rome. It was carried out following the Principles of Human Rights adopted by the World Medical Association (WMA) at the 18th WMA General Assembly, Helsinki, Finland, in June 1964, and subsequently amended by the 64th WMA General Assembly, Fortaleza, Brazil, in October 2013. We obtained written consent from all participants after fully informing them about the type and aims of the treatment. The local ethical committee approved this study (protocol N. 6279/2021).

The Inclusion criterion was the manifestation of a treatment-resistant condition during a mental disorder treated with at least one atypical antipsychotic drug or a classic neuroleptic for a previous severe treatment resistance to SGAs.

We defined TRD as an illness in which two or more antidepressant trials (adequate in doses and duration) failed, both for patients with major depressive and bipolar disorders [22]. We also defined TRS based on the failure of two or more antipsychotic trials (adequate in doses and duration) [14] and considered four weeks as an adequate trial duration [4,44]. We defined other treatment-resistant conditions on the same basis, i.e., failure of two or more antipsychotic trials in OCD, ASD, attention deficit and hyperactivity disorder (ADHD), Gilles de la Tourette syndrome (GTS), and binge-eating disorder (BED). All diagnoses were established according to DSM-5 criteria [45,46].

Exclusion criteria included minors (age < 18 years), concurrent substance use disorders (except nicotine dependence), neurological illnesses (major neurocognitive disorders, Parkinson’s disease, and Huntington’s chorea), and severe acute organic conditions (major cardiovascular pathologies, uncontrolled diabetes, severe toxic, infectious and metabolic disorders, malignancy, liver failure, and renal failure).

On these bases, all the included subjects showed a treatment-resistant mental disorder currently treated with at least one SGA, to which they were poorly responsive or unresponsive. We considered high treatment resistance (HTR), a resistant disorder currently treated with two SGAs, or clozapine, or classic neuroleptic for previous unresponsiveness to SGAs. We defined low treatment resistance (LTR) as a treatment-resistant condition currently treated with one atypical antipsychotic.

### 2.1. Genetic Analyses

According to the manufacturer’s protocol, genomic DNA was isolated from 200 μL of EDTA-anticoagulated peripheral blood samples using an automated nucleic acid extraction system (Qiasymphony, Qiagen, Hilden, Germany). We studied the following polymorphisms: *DRD2* rs1800497, rs1799732 and rs1801028, *DRD3* rs6280, and *HTR2A* rs6314, rs7997012, and rs6311. DNA polymorphisms were analyzed by Next-Generation Sequencing using the IonS5 platform (ThermoFisher Scientific, Waltham, MA, USA) and the Ion AmpliSeq™ Library Kit 2.0 reaction chemistry, following the supplier’s instructions (ThermoFisher Scientific). DNA was amplified with a pool of oligonucleotide primer pairs, each designed to amplify a genomic region of interest. Amplified DNA was used to prepare barcoded DNA libraries; clonal amplification and pooled library sequencing were performed on the IonChef/IonS5 system using the Ion 510™ and Ion 520™ and Ion 530™ Kit–Chef (ThermoFisher Scientific).

### 2.2. Statistical Analyses

We used the software IBM SPSS Statistics 27.0.1.0 (IBM Corp., Armonk, NY, USA; 1989, 2020) for all analyses, except for the Hardy–Weinberg equilibrium deviation test, for which we used an online calculator (http://apps.biocompute.org.uk/hwe-mr-calc.html, accessed on 20 March 2023) based on the methods of Rodriguez et al. [47]. Power analysis was performed using the online tool “GIGAcalculator” (https://www.gigacalculator.com/calculators/power-sample-size-calculator.php, accessed on 20 April 2023). We utilized the online tool “Carbocaption” (https://tools.carbocation.com/FDR, accessed on 13 July 2023) to perform the chi-square FDR correction for the seven comparisons of the analyzed polymorphisms.

We performed a one-sample Kolmogorov–Smirnov test to assess the normality of the distribution of the continuous variables of our study (*p* < 0.001 for each variable). Then we performed descriptive statistics with the Mann–Whitney U test for the continuous variables and the chi-square (χ^2^) test for the categorical variables applying the False Discovery Rate (FDR) correction using the Benjamini–Hochberg procedure.

We performed a binary logistic regression with the “Enter” method, using the level of drug resistance (HTR vs. LTR) as the dependent variable and the studied polymorphism genotypes as the independent, categorical variables. We included in the logistic regression only the polymorphisms for which each genotype was expressed in at least 15 participants in the whole sample, i.e., rs6311, rs6280, rs7997012, and rs1799732. We set the cut-off for statistical significance at (two-tailed) *p* < 0.05.

## 3. Results

The study participants were 129 consecutively admitted outpatients (62 women and 67 men) with a mean age of 39.22 years (SD = 16.52) affected by a treatment-resistant mental disorder currently treated with at least one antipsychotic drug prescribed by the attending psychiatrists in various settings according to pharmacological guidelines.

The whole sample included 62 patients affected by mood disorders (22 with MDD, 40 with BD), 37 with schizophrenia, and 30 with other mental disorders (20 with OCD, 4 with GTS, 3 with ASD, 2 with ADHD, and 1 with BED). 

Patients with mood disorders had a mean age of 48.35 years (SD = 16.23), those with schizophrenia 35.4 years (SD = 16.28), and other mental disorders 34.2 years (SD = 15.17). Patients with mood disorders were significantly older than patients with schizophrenia (U = 722; Z = 3.076; *p* = 0.002) and other mental disorders (U = 447; Z = 4.027; *p* < 0.001).

The HTR group included 43 patients (20 women and 23 men; mean age = 37.47, SD = 16.05), and the LTR group had 86 patients (42 women and 44 men; mean age = 40.1, SD = 16.78) without between-group differences in age (U = 1683.5; *p* = 0.408) and gender composition (*p* = 0.853).

The chi-square test showed that a diagnosis of schizophrenia, as compared to mood disorders and other mental disorders (χ^2^ = 19.464; *p* < 0.001), was more frequent in the HTR group. HTR vs. LTR patients were treated with higher doses of antipsychotic agents expressed in chlorpromazine equivalents (U = 561; *p* < 0.001).

We summarized the sociodemographic and clinical characteristics of the study sample in Table 1.

The analyzed SNPs were in Hardy–Weinberg equilibrium (rs6314 χ^2^ = 0.16; rs7997012 χ^2^ = 1.81; rs6311 χ^2^ = 1.16; rs1799732 χ^2^ = 0.98; rs1801028 χ^2^ = 1.85; rs6280 χ^2^ = 0.51), except for the rs1800497 (χ^2^ = 4.08; *p* < 0.05).

The chi-square test showed that the rs1799732 I|I vs. D|I genotype was significantly more frequent in the HTR vs. LTR group (χ^2^ = 4.159; *p* = 0.041). This finding did not remain statistically significant after adjusting for multiple comparisons (*p* = 0.287).

The power analysis showed that the study had 92.2% power (α = 0.05) to detect the observed difference (14%) between I|I and D|I frequencies between HTR and LTR groups.

We summarized the crosstab results in Table 2.

The logistic regression model we used was significant for good predictability of HTR (Omnibus Tests of Model Coefficients χ^2^ = 45.772; *p* < 0.001) and explained over 40% of the variance (Nagelkerke R^2^ = 0.415). This model proved useful, with a positive overall predictive value (79.8%).

The diagnosis of schizophrenia vs. mood disorders (OR = 22.727; B = 3.115; *p* < 0.001) and other mental disorders (OR = 12.526; B = 2.528; *p* = 0.001) significantly predicted the HTR group membership.

The *DRD3* rs6280 C|T vs. C|C genotype (OR = 22.195; B = 3.1; *p* = 0.002) and the T|T vs C|C genotype (OR = 18.47; B = 2.916; *p* = 0.003) significantly predicted the HTR group membership. In addition, the *DRD3* rs6280 C|C vs. T|T genotype inversely predicted the HTR group membership (OR = 0.054; B = −2.916; *p* = 0.003).

The *HTR2A* rs7997012 A|G vs. A|A genotype (OR = 6.859; B = 1.926; *p* = 0.046), and A|G vs. G|G genotype (OR = 2.879; B = 1.057; *p* = 0.041) significantly predicted the HTR group membership. The *HTR2A* rs6311 C|T vs. C|C genotype inversely predicted the HTR group membership with a significant trend (OR = 0.333; B = −1.1; *p* = 0.059)

Furthermore, the *DRD2* rs1799732 I|I vs. D|I genotype significantly predicted the HTR group membership (OR = 12.079; B = 2.491; *p* = 0.037) (Table 3).

## 4. Discussion

In light of a new era in which personalized medicine finds its role in a clear diagnostic-therapeutic pathway, the results of this study can enlighten some issues on whether specific polymorphism expression can predict the response to SGAs in patients with treatment resistance.

To our knowledge, this is the first study establishing the role of *DRD2* rs1799732, *DRD3* rs6280, and HTR2A rs7997012 polymorphisms in the context of resistance to antipsychotic agents in different treatment-resistant mental disorders. Previous studies had found other roles but had been conducted on samples affected by treatment-responsive mental disorders [31,48,49] or investigated the response to a specific psychopharmacological treatment only [41,50], or did not focus exclusively on samples of treatment-resistant patients [51,52].

In our sample, a diagnosis of schizophrenia is related to a greater risk of developing an HTR to SGAs than other diagnoses. Despite one may argue that resistance to antipsychotics is of paramount importance in psychotic disorders primarily, MDD, BD, OCD, GTS, ADHD, ASD, and BED may benefit from antipsychotic treatment in the cases of resistance to other medications. Since there is a lack of studies focused on SGAs treatment resistance, analyzing different DNA polymorphisms in various treatment-resistant psychiatric disorders, it is difficult to compare our findings with previous literature. Nonetheless, in a vast sea of unexplored DNA, despite current studies underlining the association of schizophrenia in particular with certain polymorphisms [53], some of which are shared with BD as well [54], it is difficult, at the moment, to understand what could be the other actors determining when a mental disease becomes treatment-resistant. In this sense, schizophrenia diagnosis seems to be associated with a higher risk of treatment resistance. We tried to take steps toward this unexplored road in light of these findings.

We showed that the rs1799732 I|I vs. D|I genotype predicted HTR membership. The rs1799732 is not responsible for structural changes in the structure of the D2 receptor, which is a 443 amino acids protein [55]. This SNP is in the promotor region, which changes transcription factor binding and may negatively influence transcription. Rs1799732 has been previously associated with nicotine dependence [56] and alcohol dependence [57], together with colorectal cancer risk [58] and increased weight gain when under antipsychotic treatment, regardless of medication [59]. Curiously, it seems to be associated with reduced harm avoidance in martial arts fighters, suggesting a role that overcomes the association with mental illness only but encompasses behavioral variants [60].

Our finding overcomes previous studies on the polymorphism’s association with schizophrenia [49], suggesting it may not play a role in the disease development but in the treatment resistance. Recent studies align with our findings, suggesting a specific pharmacodynamic variability in patients related to the rs1799732 genotype [31], which was commonly involved in the neuropathophysiology of psychoses [61]. Such a variant has also been associated with increased antipsychotic-induced parkinsonism and tardive dyskinesia in patients with schizophrenia [32], which aligns our results associating it with HTR since these two side effects are the most common neurological ADRs in the antipsychotic treatment of these patients. Serious ADRs often induce the clinician to switch medications, potentially making the patient fall into high treatment resistance. What is striking is that the variant rs1799732 has not only been associated in the literature with ADRs and pharmacodynamic variability but also with specific medication resistance (i.e., risperidone) in schizophrenia [33] and better response to olanzapine [50]. This gene variant may influence cognitive/hyperactive traits in ADHD as well, therefore affecting the disease etiology and associated co-morbid features [62], which may increase difficulty in finding a suitable medication to target all the symptomatology in ADHD, increasing the occurrence of treatment resistance, as happened in our sample. It is unclear whether the role of rs1799732 in HTR lies in a genetic predisposition to show more severe symptoms, an increased predisposition to ADRs, or a different response to antipsychotics. Still, a combination of the three mechanisms is a probable conclusion of our study, in line with current evidence, as previously explained, considering that this variant is commonly found in psychoses [61].

Another important finding is that the *DRD3* rs6280 C|T and T|T genotypes, as compared to the C|C genotype, significantly predicted a high treatment resistance to SGAs. The C|C genotype had been previously associated with schizophrenia vulnerability in the Han Chinese population [63]. Overcoming association studies and going into deep pharmacological treatment resistance, a study of 88 patients being treated for schizophrenia with olanzapine showed that those who were rs6280 (C|C) homozygotes had more significant positive symptom remission (endpoint rating of minimal or none on all PANSS clinical response positive items, 39.1%), as compared with (C|T) or (T|T) genotypes (13.8%; *p* = 0.033) [64]. Consequently, the T allele of the rs6280 can be related to a greater risk of a lower response to this kind of treatment, which is an essential component of the therapeutical approach to schizophrenia, as it is the mandatory step to which the patient should not respond to be a candidate to clozapine treatment, i.e., to be defined treatment-resistant in current medical practice [65]. As illustrated by a previous study, polymorphisms of rs6280 in the DRD3 gene have been reported to be implicated in altered dopamine binding affinity [66] and, compared with T|T homozygous patients, decreased volume and surface area in the right inferior temporal sulcus were observed in patients who were C carriers [67]. These findings suggest that C|T and T|T genotypes play a role in establishing severe disease: receptor functionality, brain structural abnormalities, and increased treatment resistance to SGAs.

Our analyses showed that the *HTR2A* rs7997012 A|G vs. other genotypes significantly predicted an HTR to antipsychotic agents. rs7997012 is a gene variation—a single-nucleotide polymorphism (SNP)—in intron 2 of the human HTR2A gene that codes for the 5-HT_2A_ receptor. The SNP varies between adenine (A) and guanine (G) DNA bases, with the G-allele being the most frequent. This is the first study to provide this finding, as this polymorphism has been previously associated with antidepressant treatment response [68,69] or proposed as a marker of citalopram response [70] but was never studied concerning antipsychotic response. Furthermore, considering that different SGAs have a relatively potent inverse agonism at 5-HT_2A_ receptors [71], and the HTR2A rs7997012 has also been involved in serotonin binding [72], the HTR to SGAs we observed might also be due to changes in the serotonin binding at 5-HT_2A_ receptors associated with this polymorphism.

We also found out that the rs6311 genotype could predict HTR to SGAs. Previous pharmacological studies suggested that the 5-HT_2A_ receptor was one of the significant pharmacological therapeutic targets for MDD. Recently, genetic studies investigated the association between the polymorphism rs6311 of the *HTR2A* gene and MDD [73]. In our findings, the *HTR2A* rs6311 C|T genotype possibly inversely predicted the HTR group membership. Furthermore, a meta-analysis suggested that rs6311, among many other polymorphisms, plays a role in the positive response to antidepressants [48], which are known to share some target receptors with many widely employed antipsychotics, such as risperidone and olanzapine.

In the context of depression, however, one may argue that HTR also associates with more severe disease symptomatology or residual symptoms after treatment, which may be the case. In addition, current evidence suggests that antipsychotics may be employed in depression, such as olanzapine, when antidepressants alone have failed [26].

### Limitations

The results of this study should be taken cautiously and need replication due to the limited sample size and real-world setting limitations. These include complex pharmacological regimes with, sometimes, long-lasting courses of mental disorders over time. Also, the sample was mixed in diagnoses, and both comparison and differences could limit the generalization of results.

## 5. Conclusions

We showed that a diagnosis of schizophrenia significantly predicted severe treatment resistance to SGAs compared to other diagnoses. Due to related pharmacodynamic variability, the *DRD2* rs1799732 I|I vs. D|I genotype, the *DRD3* rs6280 C|T and T|T vs. C|C genotypes, and the *HTR2A* rs7997012 A|G vs. other genotypes significantly predicted a severe resistance to SGAs in our sample.

Future research should establish how these findings can be used to identify patients more/less prone to developing a treatment-resistant mental disorder. This could help identify tailored intervention strategies and therapeutical approaches guided by pharmacogenomics.

## Figures and Tables

**Table 1:** Sociodemographic and clinical characteristics of the study sample.

		*N*	χ^2^			*N*	χ^2^	
Gender (f/m)	LTR	42/44	χ^2^ = 0.004 *p* = 0.95 FDR *p* = 0.95	Diagnosis (SCZ/MD/OD)	LTR	14/48/24	χ^2^ = 19.464 *p* < 0.001 * FDR *p* < 0.001 *	
	HTR	20/23			HTR	23/14/6		
	Total	62/67			Total	37/62/30		
CN (no/yes)	LTR	83/3	χ^2^ = 47.218 *p* < 0.001 * FDR *p* < 0.001 *	SGAs (no/yes)	LTR	0/86	χ^2^ = 12.585 *p* = 0.002 * FDR *p* = 0.007 *	
	HTR	18/25			HTR	6/37		
	Total	101/28			Total	6/123		
		Mean	Std. Deviation	Std. Error	U	Z	*p*	*p (FDR)*
Age, years	LTR	40.10	16.78	1.80	1683.5	−0.828	0.408	0.476
	HTR	37.47	16.05	2.44				
	Total	39.22	16.52	1.45				
Chlorpromazine equivalents, mg	LTR	220.08	225.29	27.94	561	−4.334	< 0.001 *	< 0.001 *
	HTR	492.28	340.44	56.74				
	Total	317.10	300.36	29.90				
Illness Duration, years	LTR	13.25	9.12	1.27	401	−2.622	0.009 *	0.025 *
	HTR	19.64	10.18	2.03				
	Total	15.36	9.88	1.13				

Legend. CN: classic neuroleptics; FDR = false discovery rate; HTR: high treatment resistance; LTR: low treatment resistance; MD: mood disorders; OD: other diagnoses; SCZ: schizophrenia; SGAs: second-generation antipsychotics. * Significant for *p* < 0.05.

**Table 2:** Genotype frequencies (χ^2^ crosstabs).

*DRD2* rs1800497	A|A	A|G	G|G	χ^2^	Uncorrected *p*	*p (FDR)*
LTR (%)	3 (3.5)	17 (19.8)	66 (76.7)			
HTR (%)	3 (7)	9 (20.9)	31 (72.1)			
TOT (%)	6 (4.7)	26 (20.2)	97 (75.2)	0.852	0.653	0.703
*DRD2* rs1799732	D|D	D|I	I|I	χ^2^	Uncorrected *p*	*p (FDR)*
LTR (%)	-	14 (16.3)	72 (83.7)			
HTR (%)	-	1 (2.3)	42 (97.7)			
TOT (%)	-	15 (11.6)	114 (88.4)	4.159	0.041 *	0.096
*DRD2* rs1801028	C|C	C|G	G|G	χ^2^	Uncorrected *p*	*p (FDR)*
LTR (%)	-	6 (7)	80 (93)			
HTR (%)	1 (2.3)	6 (14)	36 (83.7)			
TOT (%)	1 (0.8)	12 (9.3)	116 (89.9)	3.776	0.151	0.302
*DRD3* rs6280	C|C	C|T	T|T	χ^2^	Uncorrected *p*	*p (FDR)*
LTR (%)	14 (16.3)	37 (43)	35 (40.7)			
HTR (%)	3 (7)	19 (44.2)	21 (48.8)			
TOT (%)	17 (13.2)	56 (43.4)	56 (43.4)	2.329	0.312	0.437
*HTR2A* rs6314	A|A	A|G	G|G	χ^2^	Uncorrected *p*	*p (FDR)*
LTR (%)	-	19 (22.1)	67 (77.9)			
HTR (%)	1 (2.3)	10 (23.3)	32 (74.4)			
TOT (%)	1 (0.8)	29 (22.5)	99 (76.7)	2.063	0.357	0.437
*HTR2A* rs7997012	A|A	A|G	G|G	χ^2^	Uncorrected *p*	*p (FDR)*
LTR (%)	12 (14)	28 (32.6)	46 (53.5)			
HTR (%)	3 (7)	19 (44.2)	21 (48.8)			
TOT (%)	15 (11.6)	47 (36.4)	67 (51.9)	2.383	0.304	0.437
*HTR2A* rs6311	C|C	C|T	T|T	χ^2^	Uncorrected *p*	*p (FDR)*
LTR (%)	19 (22.1)	48 (55.8)	19 (22.1)			
HTR (%)	15 (34.9)	17 (39.5)	11 (25.6)			
TOT (%)	34 (26.4)	65 (50.4)	30 (23.3)	3.437	0.179	0.313

Legend. FDR: false discovery rate; HTR: high treatment resistance; LTR: low treatment resistance. * Significant for *p* < 0.05.

**Table 3:** Logistic regression model description and results.

		χ^2^	df	*p*	Model Summary	−2 Log Likelihood	Nagelkerke R^2^		
**Omnibus Tests of Model Coefficients**		45.772	11	<0.001 *		118.449	0.415		
Hosmer and Lemeshow Test		9.240	8	0.322					
		B	S.E.	Wald	df	*p*	OR	95% CI for OR	
								Lower	Upper
a	Age	−0.004	0.015	0.053	1	0.818	0.996	0.967	1.027
	Sex (female)	0.578	0.490	1.390	1	0.238	1.783	0.682	4.661
	Diagnosis			20.965	2	<0.001 *			
	Schizophrenia	2.528	0.734	11.846	1	0.001 *	12.526	2.969	52.845
	Mood disorders	−0.587	0.685	0.733	1	0.392	0.556	0.145	2.131
	*HTR2A* rs6311			3.648	2	0.161			
	*HTR2A* rs6311 C|C	0.823	0.659	1.559	1	0.212	2.277	0.626	8.287
	*HTR2A* rs6311 C|T	−0.277	0.597	0.215	1	0.643	0.758	0.235	2.443
	*DRD3* rs6280			10.274	2	0.006 *			
	*DRD3* rs6280 C|C	−2.916	0.965	9.137	1	0.003 *	0.054	0.008	0.359
	*DRD3* rs6280 C|T	0.184	0.509	0.130	1	0.718	1.202	0.443	3.258
	*HTR2A* rs7997012			6.082	2	0.048 *			
	*HTR2A* rs7997012 A|A	−0.868	0.927	0.878	1	0.349	0.420	0.068	2.580
	*HTR2A* rs7997012 A|G	1.057	0.517	4.188	1	0.041 *	2.879	1.046	7.927
	*DRD2* rs1799732 D|I	−2.491	1.195	4.347	1	0.037 *	0.083	0.008	0.861
	Constant	−1.427	0.985	2.097	1	0.148	0.240		
		B	S.E.	Wald	df	*p*	OR	95% CI for OR	
								Lower	Upper
b	Age	−0.004	0.015	0.053	1	0.818	0.996	0.967	1.027
	Sex (male)	−0.578	0.490	1.390	1	0.238	0.561	0.215	1.467
	Diagnosis			20.965	2	<0.001 *			
	Mood disorders	−3.115	0.706	19.463	1	<0.001 *	0.044	0.011	0.177
	Other mental disorders	−2.528	0.734	11.846	1	0.001 *	0.080	0.019	0.337
	*HTR2A* rs6311			3.648	2	0.161			
	*HTR2A* rs6311 C|T	−1.100	0.581	3.578	1	0.059	0.333	0.107	1.041
	*HTR2A* rs6311 T|T	−0.823	0.659	1.559	1	0.212	0.439	0.121	1.598
	*DRD3* rs6280			10.274	2	0.006 *			
	*DRD3* rs6280 C|T	3.100	0.985	9.912	1	0.002 *	22.195	3.222	152.873
	*DRD3* rs6280 T|T	2.916	0.965	9.137	1	0.003 *	18.470	2.788	122.362
	*HTR2A* rs7997012			6.082	2	0.048 *			
	*HTR2A* rs7997012 A|G	1.926	0.966	3.974	1	0.046 *	6.859	1.033	45.550
	*HTR2A* rs7997012 G|G	0.868	0.927	0.878	1	0.349	2.383	0.388	14.645
	*DRD2* rs1799732 I|I	2.491	1.195	4.347	1	0.037 *	12.079	1.161	125.663
	Constant	−3.774	1.860	4.116	1	0.042 *	0.023		

a: Reference categories. Sex: male; Diagnosis: other mental disorders; rs6311: T|T; rs6280: T|T; rs7997012: G|G; rs1799732: I|I. b: Reference categories. Sex: Female; Diagnosis: Schizophrenia; rs6311: C|C; rs6280: C|C; rs7997012: A|A; rs1799732: D|I. Legend: df: degrees of freedom; OR: Odds ratio; CI: confidence interval; * Significant for *p* < 0.05.

## Data Availability

The research data can be requested from the first authors (A.D.C. and M.S.).

## Abbreviations

ADHD: attention deficit and hyperactivity disorder; ADRs: adverse drug reactions; APA: American Psychiatric Association; ASD: autism spectrum disorders; BD: bipolar disorder; BED: binge-eating disorder; CANMAT: Canadian Network for Mood and Anxiety Treatment; CPZ: chlorpromazine; DRD2: dopamine receptor D_2_ gene; DSM: Diagnostic and Statistical Manual of Mental Disorders; EPS: extra-pyramidal symptoms; FDA: Food and Drug Administration; FGAs: first-generation antipsychotics; GTS: Gilles de la Tourette Syndrome; HTR: high treatment resistance; HTR2A: serotonin receptor 5-HT_2A_ gene; LTR: low treatment resistance; MDD: major depressive disorder; NICE: The National Institute for Health and Care Excellence; OCD: obsessive–compulsive disorder; PGx: pharmacogenomics; PTSD-SP: post-traumatic stress disorder with psychotic features; SGAs: second-generation antipsychotics; SNPs: single-nucleotide polymorphisms; SSD: schizophrenia spectrum disorders; TRD: treatment-resistant depression; TRRIP: Treatment Response and Resistance in Psychosis Working Group Consensus; TRS: treatment-resistant schizophrenia; WMA: World Medical Association.
